# Biospeciation of Potential Vanadium Drugs of Acetylacetonate in the Presence of Proteins

**DOI:** 10.3389/fchem.2020.00345

**Published:** 2020-05-07

**Authors:** Giuseppe Sciortino, Valeria Ugone, Daniele Sanna, Giuseppe Lubinu, Simone Ruggiu, Jean-Didier Maréchal, Eugenio Garribba

**Affiliations:** ^1^Dipartimento di Chimica e Farmacia, Università di Sassari, Sassari, Italy; ^2^Departament de Química, Universitat Autònoma de Barcelona, Cerdanyola del Vallés, Barcelona, Spain; ^3^Istituto di Chimica Biomolecolare, Consiglio Nazionale delle Ricerche, Sassari, Italy

**Keywords:** metal drugs, vanadium, anticancer action, antidiabetic action, proteins, transport in the organism, drug design

## Abstract

Among vanadium compounds with potential medicinal applications, [V^IV^O(acac)_2_] is one of the most promising for its antidiabetic and anticancer activity. In the organism, however, interconversion of the oxidation state to +III and +V and binding to proteins are possible. In this report, the transformation of V^III^(acac)_3_, V^IV^O(acac)_2_, and V^V^O_2_(acac)${}_{2}^{-}$ after the interaction with two model proteins, lysozyme (Lyz) and ubiquitin (Ub), was studied with ESI-MS (ElectroSpray Ionization-Mass Spectroscopy), EPR (Electron Paramagnetic Resonance), and computational (docking) techniques. It was shown that, in the metal concentration range close to that found in the organism (15–250 μM), V^III^(acac)_3_ is oxidized to V^IV^O(acac)^+^ and V^IV^O(acac)_2_, which—in their turn—interact with proteins to give *n*[V^IV^O(acac)]–Protein and *n*[V^IV^O(acac)_2_]–Protein adducts. Similarly, the complex in the +IV oxidation state, V^IV^O(acac)_2_, dissociates to the mono-chelated species V^IV^O(acac)^+^ which binds to Lyz and Ub. Finally, V^V^O_2_(acac)${}_{2}^{-}$ undergoes complete dissociation to give the 'bare' V^V^O${}_{2}^{+}$ ion that forms adducts *n*[V^V^O_2_]–Protein with *n* = 1–3. Docking calculations allowed the prediction of the residues involved in the metal binding. The results suggest that only the V^IV^O complex of acetylacetonate survives in the presence of proteins and that its adducts could be the species responsible of the observed pharmacological activity, suggesting that in these systems V^IV^O^2+^ ion should be used in the design of potential vanadium drugs. If V^III^ or V^V^O_2_ potential active complexes had to be designed, the features of the organic ligand must be adequately modulated to obtain species with high redox and thermodynamic stability to prevent oxidation and dissociation.

## Introduction

Among the metal-based drugs, vanadium (V) compounds have drawn a growing interest for their pharmacological properties shown both *in vivo* and *in vitro* studies. In particular, several V complexes have been proposed as antidiabetic and anticancer potential drugs (Sakurai et al., 2008; Costa Pessoa et al., 2015a; Kioseoglou et al., 2015; Rehder, 2016) and some of them reached the clinical trials, for example bis(ethylmaltolato)oxidovanadium(IV) or BEOV (Thompson and Orvig, 2006; Thompson et al., 2009). Over the last years, many complexes were studied with vanadium in one of the three oxidation states found in biological systems, +III, +IV, and +V. However, the mechanism of action and active oxidation state in the organism remain unclear and biospeciation has not been completely ascertained. This lack of knowledge is one of the reasons why V compounds are not considered for clinical tests by pharmaceutical companies and limits the possibility of a rational design of new potential drugs with more activity and lower toxicity than those proposed up to now (Scior et al., 2016).

Among these compounds, bis(acetylacetonato)-oxidovanadium(IV) or V^IV^O(acac)_2_ is one of the most studied (Makinen and Brady, 2002; Crans et al., 2018). It favors the decrease of blood glucose concentration and is more effective than the inorganic salt V^IV^OSO_4_ in the suppression of hepatic glycolysis in streptozotocin-induced diabetic rats, and this effect cannot be related only to an improved intestinal absorption (Reul et al., 1999; Amin et al., 2000). V^IV^O(acac)_2_ enhances in cells the IR (insulin receptor) kinase activity more than other V^IV^O chelate species (Makinen and Salehitazangi, 2014), even though it has not been proven if its action is direct through the stimulation of the IR kinase activity or indirect through the activation of other tyrosine kinases or inhibition of tyrosine phosphatases, for example PTP-1B (Ou et al., 2005; Makinen et al., 2007). V^IV^O(acac)_2_ exhibits also important anti-proliferative effects: it blocks the G1/S phase of the cell cycle progression via an activated ERK (extracellular signal-regulated kinase) signal in human hepatoma HepG2 cells (Fu et al., 2008), while in human pancreatic cancer cell line AsPC-1 it induces cell cycle arrest at the G2/M phase and causes an increase of ROS (reactive oxygen species) concentration (Wu et al., 2016). These results indicate that V-based complexes may be potentially applied as antidiabetic and anticancer agents to treat patients suffering from both the diseases (Liu et al., 2013).

Species related to V^IV^O(acac)_2_ in the organism are V^III^(acac)_3_ and V^V^O_2_(acac)${}_{2}^{-}$. In fact, on one hand several experimental evidences showed that V^IV^ complexes can be rapidly oxidized to V^V^ in the cell culture medium and, on the other, that +IV state can converge to +III in the gastrointestinal medium, in the blood and reducing cellular environment; for these reasons, more than one species could contribute to the global biological action. The biotransformation of V^IV^O(acac)_2_ is obviously related to the pharmacological efficacy, since it determines the gastrointestinal absorption, the transport in blood and—overall—the availability of the active species. The interaction with proteins plays a key role in the biospeciation of a potential metal-based drug in the organism due to their high concentration and/or high affinity toward the metals (Costa Pessoa et al., 2015b).

In this paper, the transformation of V^III^(acac)_3_, V^IV^O(acac)_2_, and V^V^O_2_(acac)${}_{2}^{-}$ in the presence of two model proteins, lysozyme (Lyz) and ubiquitin (Ub), was studied by ESI-MS (ElectroSpray Ionization-Mass Spectrometry), EPR (Electron Paramagnetic Resonance), and computational (docking and QM) techniques. Both ESI-MS and EPR techniques present, for what concerns this work, pros and cons. The main advantage of ESI-MS is that the metal concentrations used for experiments are in the range 1–100 μM, i.e., that found for V at physiological conditions (Jakusch and Kiss, 2017); the limitation is that it does not give any information on the structure of the revealed vanadium adducts and that the intensity of the peaks cannot be related to the amount of the species in solution. With regard to EPR spectroscopy, it allows only the recognition of the type of amino acid donors involved in the metal coordination (His-N, Asp/Glu-COO, or carbonyl-CO, etc.), with the limitation that the specific site and its three-dimensional structure cannot be identified (Chasteen, 1981; Smith et al., 2002).

The results of this study on the transformation of V^IV^O(acac)_2_ and—in general—of bis-chelated complexes with stoichiometry V^IV^OL_2_, where L is a bidentate monoanionic organic ligand, could be very useful to ascertain the active species in the organism and be used as a guide for a rational design of new potential V drugs.

## Materials and Methods

### Chemicals

Water was deionized through the purification system Millipore MilliQ Academic or purchased from Sigma-Aldrich (LC-MS grade). V^III^(acac)_3_ and V^IV^O(acac)_2_, NH_4_V^V^O_3_, acetylacetone (Hacac), and 4-(2-hydroxyethyl)piperazine-1-ethanesulfonic acid (HEPES) were Sigma-Aldrich products of the highest grade available and used as received.

Chicken egg white lysozyme (Lyz; molecular mass of 14.3 kDa, code 62970) and ubiquitin from bovine erythrocytes (Ub; molecular mass of 8.6 kDa, code U6253) were purchased from Sigma-Aldrich.

### ESI-MS Measurements

The solutions for ESI-MS measurements were prepared as follows. Briefly, the solid complexes V^III^(acac)_3_ and V^IV^O(acac)_2_ or NH_4_V^V^O_3_ plus Hacac in molar ratio 1/2 were dissolved in LC-MS grade water to obtain a V concentration of 1.0–2.0 mM. Subsequently, all the solutions were diluted in LC-MS grade water with an aliquot of a stock protein solution (500 μM) to reach (V complex)/Protein ratios of 3/1, 5/1, and 10/1 and a protein concentration of 5 μM. In all the solutions pH was in the range 6–7. Immediately after the preparation of the solutions, ESI-MS spectra were acquired.

Mass spectra in the positive-ion or negative-ion mode were recorded on a Q Exactive™ Plus Hybrid Quadrupole-Orbitrap™ (Thermo Fisher Scientific) mass spectrometer. The solutions were infused at a flow rate of 5.00 μL/min into the ESI chamber. The spectra were obtained in the m/z range 300–4,500 at a resolution of 140,000 and accumulated for at least 5 min to increase the signal-to-noise ratio. The instrumental setting for the measurement of the spectra in positive-ion mode were: spray voltage 2,300 V, capillary temperature 250°C, sheath gas 10 (arbitrary units), auxiliary gas 3 (arbitrary units), sweep gas 0 (arbitrary units), probe heater temperature 50°C. The setting used for negative-ion mode spectra were: spray voltage −1,900 V, capillary temperature 250°C, sheath gas 20 (arbitrary units), auxiliary gas 5 (arbitrary units), sweep gas 0 (arbitrary units), probe heater temperature 14°C. ESI-MS spectra were analyzed by using Thermo Xcalibur 3.0.63 software (Thermo Fisher Scientific) and the average deconvoluted monoisotopic masses were obtained through the Xtract tool integrated in the software.

### EPR Measurements

EPR spectra were acquired by dissolving in ultra-pure water V^III^(acac)_3_ to obtain a V^III^ concentration of 1 mM. To the solutions, HEPES buffer of 0.1 M concentration was added. The value of pH was brought in the pH range 5–7 and a protein (Lyz or Ub) was added to 1 mL of the solution with the V species, to reach a concentration of 0.5 mM and a molar ratio (V complex)/Protein of 2/1. Subsequently, an aliquot of the solution with V complex was added to bring the ratio (V complex)/Protein to 3/1.

EPR spectra were recorded at 120 K with an X-band Bruker EMX spectrometer equipped with a HP 53150A microwave frequency counter using a microwave frequency of 9.40–9.41 GHz, microwave power of 20 mW, time constant of 81.92 ms, modulation frequency of 100 kHz, modulation amplitude of 0.4 mT, and resolution of 4,096 points. The spectra were immediately recorded after the preparation of the samples. To increase the signal to noise ratio, signal averaging was employed (Sanna et al., 2009).

### DFT and Docking Calculations

All the DFT calculations were performed with Gaussian 09 (revision D.01) (Frisch et al., 2010). The geometries and harmonic frequencies of V^IV^O and V^V^O_2_ complexes were computed at the level of theory B3P86/6-311++g(d,p) with the SMD model (Marenich et al., 2009) for water.

Docking calculations were carried out with GOLD 5.8 software (Jones et al., 1997) on the X-ray structure available in the Protein Data Bank (PDB) of lysozyme [PDB code: 2LYZ (Diamond, 1974)] and ubiquitin [3H1U (Qureshi et al., 2009)]. The PDB structure was cleaned removing all the small molecules and crystallographic waters, and hydrogen atoms were added with the UCSF Chimera (Pettersen et al., 2004).

The docking simulations were carried out constructing in the region of interest an evaluation sphere of 12 Å. The *coordination* docking calculations (with an *active* binding V–donor) were performed on the DFT optimized moieties V^IV^O(acac)^+^ and *cis*-V^V^O${}_{2}^{+}$, activating one, two or three equatorial coordination positions, respectively, with dummy hydrogen atoms (Sciortino et al., 2017, 2018a,b, 2019a). All the possible moieties V^V^O_2_(H_2_O)_*n*_^+^ with *n* = 0–2 were taken into account during dockings and the proposed solutions in the text are those with higher scoring. For *coordination* dockings the regions with the potential coordinating residues were examined with the GOLD rotamers libraries (Lovell et al., 2000). For the *non-coordination* dockings (*inert* binding V···donor) with V^IV^O(acac)_2_, the whole rigid protein was considered. The solutions were analyzed by means of GaudiView (Rodríguez-Guerra Pedregal et al., 2017).

To identify potential binding sites, the protein space was first probed with multisite.py for zones where at least two potential coordinating residues (Asp, Glu, Asn, Gln, and His) featured an α- and a β-carbon within a range of distances from each queried grid point of 2.6–6.4 and 3.4–7.2 Å, respectively (Sciortino et al., 2019b). Both the protonation states at δ and ε nitrogens of His imidazole ring were taken into account during the simulations.

Genetic algorithm (GA) parameters have been set to 50 GA runs and a minimum of 100,000 operations. The other parameters of GA were set to default.

The scoring (*Fitness* of GoldScore) was calculated with the modified version of GoldScore scoring function, which was validated in previously published papers (Sciortino et al., 2017, 2018a, 2019a). The best solutions (binding poses) were evaluated through the mean (*F*_mean_) and the highest value (*F*_max_) of the *Fitness* associated with each pose, the population of the cluster containing the best pose and the position in the *Fitness* ranking of the computed cluster.

## Results and Discussion

### Interaction of V^III^(acac)_3_ and V^IV^O(acac)_2_ With Proteins

The species distribution diagram of the V^III^/acac system with molar ratio 1/3 and V concentration of 150 μM, that does not account for the oxidation process to V^IV^, can be built using the data of the thermodynamic stability constants reported in the literature (Brito et al., 2009). The diagram is shown in Figure S1 of the Supplementary Material and suggests that the main species in the pH range 6.0–7.0 is V^III^(acac)_3_. ESI-MS spectrum was recorded on V^III^(acac)_3_ at pH 6.2 in a degassed aqueous solution to evaluate its stability in the absence of a protein (Figure S2). The results indicate that the oxidation to V^IV^O is only partial: even though the intensity of the MS signal cannot be directly related to the concentration in solution, the peaks of the adducts [V^III^(acac)_3_+H^+^] and [V^III^(acac)_3_+Na^+^] (m/z = 349.09 and 371.07, respectively) are much more intense than those assigned to [V^IV^O(acac)_2_+H^+^] and [V^IV^O(acac)_2_+Na^+^] (m/z = 266.04 and 288.02). The simulation of the two peaks of V^III^(acac)_3_ is shown in Figures S3, S4 and confirms the attribution.

**Figure 1:**
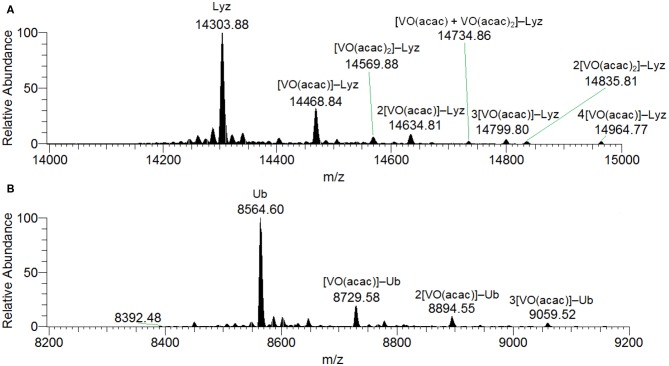
Deconvoluted ESI-MS spectrum recorded on the systems: **(A)** V^III^(acac)_3_ and lysozyme, pH 6.2, and **(B)** V^III^(acac)_3_ and ubiquitin, pH 6.3. The molar ratio V^III^/Protein was 3/1 and Protein concentration 50 μM. Under these experimental conditions, V^III^ species fully oxidizes to V^IV^O.

When the spectra were measured on the system V^III^(acac)_3_/Lyz the transformation to V^IV^O is complete and no peaks assignable to the *active* or *inert* binding of V^III^(acac)_3_ to the protein could be revealed. With a molar ratio of 3/1 and a protein concentration of 5 μM, the peaks of the free protein at 14304 Da plus the signals of the adducts *n*[V^IV^O(acac)]–Lyz with *n* = 1–4 (with mass of 14469, 14635, 14800 and 14965 Da, respectively) were observed (Figure S5). For these species the equatorial interaction with one or two protein residues is possible. Similar results were obtained at (V complex)/Protein ratio of 5. When higher protein concentration was used (50 μM), the spectra show a series of peaks belonging to *n*[V^IV^O(acac)]–Lyz, with *n* = 1–4, while the signals at 14570 and 14836 Da can be attributed to [V^IV^O(acac)_2_]–Lyz and 2[V^IV^O(acac)_2_]–Lyz (Figure 1A). Moreover, the adduct [V^IV^O(acac)+V^IV^O(acac)_2_]–Lyz at 14735 Da is detected with the contemporaneous coordination of mono- and bis-chelated moieties (Figure 1A). Therefore, the increase of the V concentration favors the interaction of the bis-chelated complex V^IV^O(acac)_2_ with lysozyme, even though the binding of the fragment VO(acac)^+^ appears to be favored compared to V^IV^O(acac)_2_. Concerning the interaction between [VO(acac)_2_] with lysozyme, no equatorial sites are accessible and so only two possibilities exist: an *inert* binding with the protein surface or a weak axial *active* binding through an amino acid residue.

**Figure 2:**
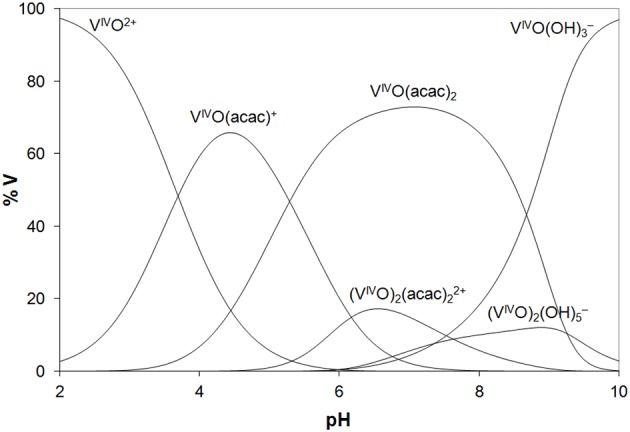
Distribution diagram of the species as a function of pH in a solution containing V^IV^O^2+^ ion and acac (V^IV^O/acac = 1/2, V^IV^O = 1.50 ×10^−4^ M). The stability constants of the V^IV^O–acac complexes were taken from Crans et al. (2001) and of the hydrolytic species from Buglyo et al. (2000).

In the analysis of the results on the system V^IV^O(acac)_2_/Lyz, it must be considered that in the examined pH range V^IV^O(acac)_2_ is the major species in aqueous solution and that it coexists with 1:1 complex V^IV^O(acac)^+^. The diagram with the distribution of the species is represented in Figure 2. The ESI-MS spectra are in agreement with these data and show similar results with formation of the adducts *n*[V^IV^O(acac)]–Lyz and *n*[V^IV^O(acac)_2_]–Lyz (Figure S6).

**Figure 3:**
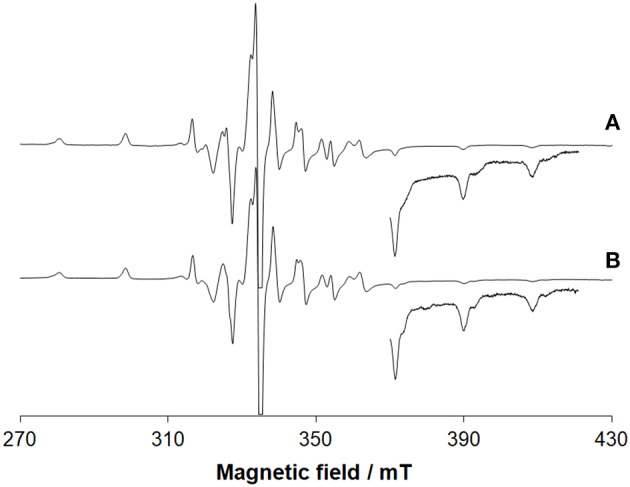
EPR spectra recorded on frozen solutions (120 K) of the systems: **(A)** V^III^(acac)_3_ and lysozyme and **(B)** V^III^(acac)_3_ and ubiquitin. V^III^/Protein molar ratio was 3/1 and V concentration 1 mM. pH was 5.70 and 5.15, respectively. The region at high field was amplified 10 times. At these conditions, V^III^ species fully oxidizes to V^IV^O.

EPR spectroscopy confirms the oxidation of V^III^ and the resonances due to the V^IV^O ion (3*d*^1^ electronic configuration) are revealed (Figure 3A). Beside the absorption due to V^IV^O(acac)_2_ not covalently bound to lysozyme, the signals were attributed to [V^IV^O(acac)]–Lyz adducts with the equatorial binding of two protein residues (*g*_z_ ~ 1.943 and *A*_z_ ~ 171–172 × 10^−4^ cm^−1^). This is illustrated in Figures S7A,B.

The deconvoluted ESI-MS spectrum of the system V^III^(acac)_3_/Ub 3/1 using an Ub concentration of 5 or 50 μM (Figure 1B) showed the presence of [V^IV^O(acac)]–Ub, 2[V^IV^O(acac)]–Ub and 3[V^IV^O(acac)]–Ub. This means that three moieties V^IV^O(acac)^+^ bind to ubiquitin, which replaces two weak water ligands in the two adjacent equatorial positions of V^IV^O(acac)(H_2_O)${}_{2}^{+}$. When the interaction between V^IV^O(acac)_2_ and ubiquitin is considered, the results are similar to those revealed with V^III^ and recently published, that indicate the formation of the adducts *n*[V^IV^O(acac)]–Ub, with *n* = 1–3 (Ugone et al., 2019). For the system containing Ub as well, EPR measurements indicated the complete transformation of V^III^(acac)_3_ to V^IV^O(acac)^+^ moiety, which in solution interacts with ubiquitin (Figure 3B).

Overall, the behavior of the V^III^ and V^IV^O systems with the two model proteins is very similar. The results indicate that, in the metal concentration range 15–250 μM, i.e., that found in the organism (Jakusch and Kiss, 2017), V^III^(acac)_3_ is quantitatively oxidized to V^IV^O(acac)^+^ and V^IV^O(acac)_2_, which—in their turn—interact with proteins. Compared to the binary systems without proteins, the oxidation is favored probably due to the stabilization of the V^IV^O adducts upon the binding with the amino acid residues. So, the results obtained with V^III^ can be interpreted considering the species distribution diagram shown in Figure 2 for V^IV^O–acac system, postulating that the oxidation process in the presence of the proteins is quantitative. The reactions which account for the formation of the adducts [V^IV^O(acac)^+^]–Protein and [V^IV^O(acac)_2_]–Protein are as follows:

(1) $$\begin{array}{r} {\text{V}}^{\text{III}}{\left(\text{acac}\right)}_{3}+{\text{H}}_{2}\text{O}+\text{Protein}\to \\ {}[{\text{V}}^{\text{IV}}{\text{O}\left(\text{acac}\right)}^{+}\text{]–Protein}+2\text{Hacac}+{\text{e}}^{-} \end{array}$$

(2) $$\begin{array}{r} {\text{V}}^{\text{III}}{\left(\text{acac}\right)}_{3}+{\text{H}}_{2}\text{O}+\text{Protein}\to \\ {}[{\text{V}}^{\text{IV}}{\text{O}\left(\text{acac}\right)}_{2}\text{]–Protein}+\text{Hacac}+{\text{H}}^{+}+{\text{e}}^{-} \end{array}$$

The number of V^IV^O(acac)^+^ and V^IV^O(acac)_2_ moieties bound depends on the vanadium concentration and type of protein; in particular, the binding of V^IV^O(acac)_2_ is favored with increasing V concentration and going from Ub to Lyz.

**Figure 4:**
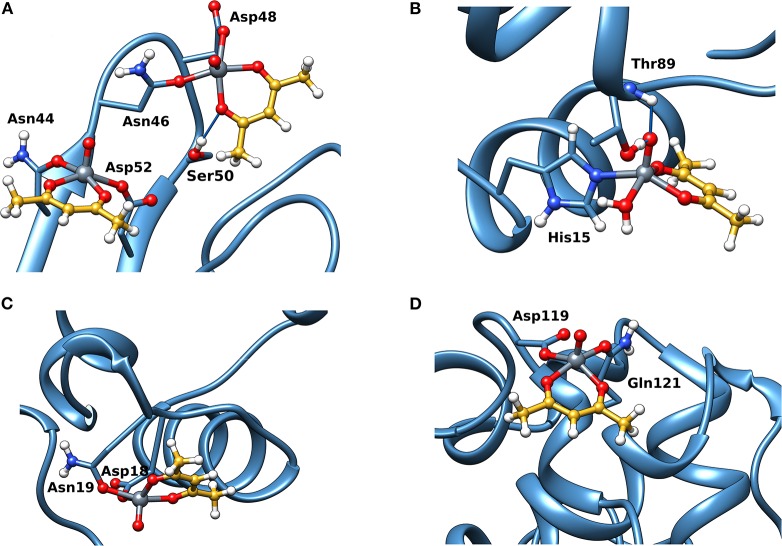
Most stable structures obtained by docking methods for the *active* binding of V^IV^O(acac)^+^ to Lyz: **(A)** site **A** with (Asn44, Asp52) and (Asn46, Asp52); **(B)** site **B** with His15; **(C)** site **C** with (Asp18, Asn19); and **(D)** site **D** with (Asp119, Gln121). The hydrogen bonds are indicated with the full blue lines.

**Figure 5:**
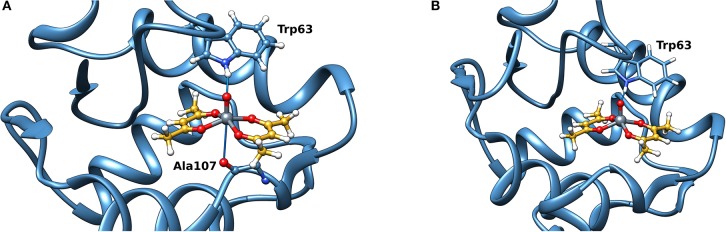
Most stable site obtained by docking methods for the *inert* binding of V^IV^O(acac)_2_ to Lyz: **(A)** V=O**···**HN–Trp63 is 2.029 Å and V**···**OCN–Ala107 3.370 Å and **(B)** V=O**···**HN–Trp63 is 1.644 Å and V**···**OCN–Ala107 3.875 Å. The weak bonds with Trp63 and Ala107 are indicated with the full blue lines.

Docking calculations were performed to confirm ESI-MS and EPR findings and get information on the residues involved in the V^IV^ binding. For lysozyme, the results indicate that four-five sites are available for V^IV^O(acac)^+^ interaction, in agreement with ESI-MS measurements (Table 1 and Figure 4). In the region 44–52 one or two V^IV^O(acac)^+^ can bind to protein with the simultaneous coordination of two residues. The affinity order is as follows: (Asn46, Asp48) > (Asn46, Asp52) ~ (Asn44, Asp52), the *F*_mean_ values being in the range 35.9–40.8 with a population between 41/50 and 50/50. In all the three sites, named **A**, the donor set is (NCO, COO^−^) and this accounts for the *A*_z_ value measured in the EPR spectra (*A*_z_ ~ 171–172 × 10^−4^ cm^−1^, see above). It must be observed that the contemporaneous coordination to both (Asn46, Asp52) and (Asn44, Asp52) donor set is not compatible and the binding to the first site excludes the second one. The second site (site **B**) is located in the *N*-terminal region, where His15 plus a water ligand are coordinated to the V^IV^O^2+^ ion, and a hydrogen bond between V=O group and Thr89 stabilizes the binding. Interestingly, the His15 equatorial binding is not expected for bis-chelated *cis* moieties such as *cis*-V^IV^O(picolinato)_2_ or *cis*-V^IV^O(maltolato)_2_ due to the steric hindrance of the second ligand with an (equatorial-axial) arrangement (Sciortino et al., 2017). The third and fourth sites are weaker than these ones and are based on the coordination of (Asp18, Asn19) with *F*_mean_ = 30.9 (site **C**) and (Asp119, Gln121) with *F*_mean_ = 30.7 (site **D**). The four identified sites are represented in Figure 4. For the interaction of V^IV^O(acac)_2_, only *inert* binding must be considered: the best site is based on a hydrogen bond stabilization between the V^IV^=O and NH group of Trp63 residue and a weak axial interaction of NCO of Ala107 with vanadium. The two solutions are displayed in Figures 5A,B, showing that with increasing the strength of the axial interaction with Ala107 decreases the strength of the contact of V=O with Trp63 and *viceversa*; in Figure 5A V=O**···**HN–Trp63 is 2.029 Å and V**···**OCN–Ala107 is 3.370 Å, while in Figure 5B V=O**···**HN–Trp63 is 1.644 Å and V**···**OCN–Ala107 is 3.875 Å.

**Table 1:** Docking solutions for the active binding of the V^IV^O(acac)^+^ moiety with Lyz and Ub.

**Protein**	**Site**	**Residues**	**V–donor^a^**	**2nd interaction**	**${\text{F}}_{\text{max}}^{\text{b}}$**	**${\text{F}}_{\text{mean}}^{\text{c}}$**	**Pop.^d^**	**Rank.^e^**
Lyz	A	Asn46, Asp48	2.082, 2.255	O_acac_···Ser50	41.8	40.8	50/50	I
	A	Asn44, Asp52	2.315, 2.117	–	37.1	35.9	45/50	I
	A	Asn46, Asp52	2.279, 2.306	–	37.4	36.3	41/50	II
	B	His15	2.351	VO···Thr89	40.5	38.7	8/50	I
	C	Asp18, Asn19	2.134, 2.179	–	32.1	30.9	49/50	I
	D	Asp119, Gln121	2.162, 1.946	–	33.7	30.7	38/50	I
Ub	1	Glu16, Glu18	2.300, 2.342	–	46.2	44.3	7/50	I
	1'	Glu18, Asp21	2.382, 2.236	–	42.8	39.3	10/50	II
	2	Glu24, Asp52	2.328, 2.353	–	38.2	37.2	44/50	I
	2'	Glu51, Asp52	2.373, 2.373	–	36.3	34.9	6/50	II

a *Distance between V and protein donors in Å*.

b *GoldScore Fitness value obtained for the more stable pose of each cluster (F_max_)*.

c *Mean value of GoldScore Fitness for each cluster (F_mean_)*.

d *Population of the cluster*.

e *Ranking of the identified cluster*.

**Figure 6:**
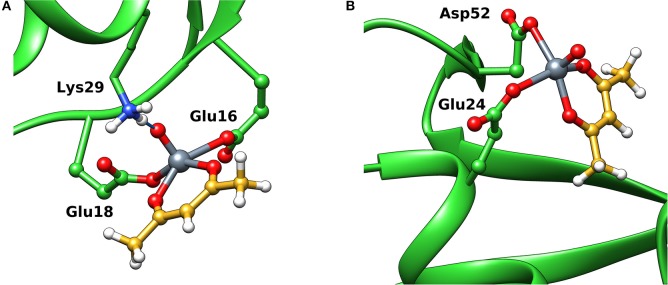
Most stable structures obtained by docking methods for the *active* binding of V^IV^O(acac)^+^ to Ub: **(A)** site **1** with (Glu16, Glu18) and **(B)** site **2** with (Glu24, Asp52). The hydrogen bonds are indicated with the full blue lines.

When the interaction of V^IV^O species and ubiquitin is examined, the data indicated that two main sites exist. The first is based on the coordination of (Glu 16, Glu18; site **1**) or (Glu18, Asp21; site **1'**) (Ugone et al., 2019); the two sites are mutually exclusive and have *F*_mean_ in the range 39.3–44.3 with population between 14 and 20%. The second site is due to the equatorial binding of the couple (Glu24, Asp52; site **2**) or (Glu51, Asp52; site **2'**) (Ugone et al., 2019); in this case too, these sites are not independent with *F*_mean_ = 34.9–37.2 and population = 12–88%. The two sites are shown in Figure 6 and the values of *F*_max_, *F*_mean_ and population in Table 1.

### Interaction of V^V^O_2_(acac)${}_{2}^{-}$ With Proteins

Positive-ion mode and negative-ion mode ESI-MS spectra were recorded on the system H_2_V^V^O${}_{4}^{-}$/Hacac 1/2 with V concentration of 150 μM at pH 7.0 (Figure S8). In the literature, the thermodynamic stability constants on the system V^V^/acac are lacking. The data for maltol, a (O,O) ligand with similar basicity [p*K*_a_ is 8.44 for maltol (Buglyo et al., 2002) and 8.76 for acetylacetone (Crans et al., 2001)], indicate that at these experimental conditions, the percentage of V in solution as V^V^O_2_(maltolato)${}_{2}^{-}$ in the pH range 6–7 is <30% (Elvingson et al., 1996); the amount of V^V^O_2_(maltolato)${}_{2}^{-}$ increases significantly with V concentration and overcomes 70% when it is ten times larger, i.e., 1.5 mM. This demonstrates that the hydrolytic processes cannot be neglected with decreasing V concentration. A similar behavior is expected for V^V^-acac system: specifically, the complex V^V^O_2_(acac)${}_{2}^{-}$ is formed only at high V concentration (around some mM), but it is not stable when the metal concentration is in the order of μM.

**Figure 7:**
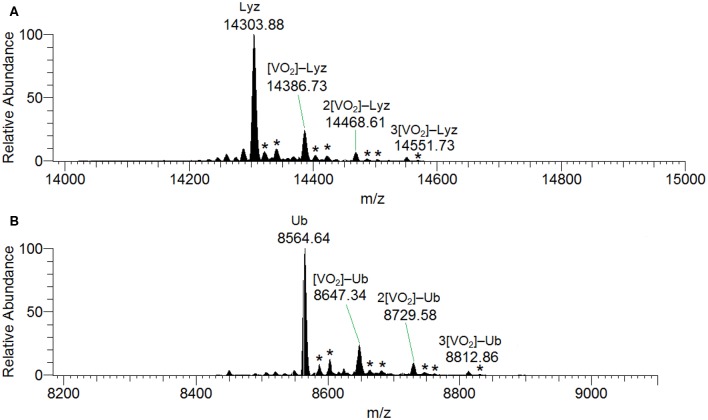
Deconvoluted ESI-MS spectrum recorded on the systems containing: **(A)** V^V^O_2_(acac)${}_{2}^{-}$ and lysozyme, pH 6.4, and **(B)** V^V^O_2_(acac)${}_{2}^{-}$ and ubiquitin, pH 6.4. The molar ratio V^III^/Protein was 3/1 and Protein concentration 50 μM. With the asterisks the adducts with the binding of one and two water molecules are indicated.

ESI-MS results indicated the presence of [Hacac+H^+^] and [Hacac+Na^+^] in the positive-ion mode (Figure S8A), and of [H_2_V^V^O${}_{4}^{-}$] in the negative-ion mode (Figure S8B), derived from the hydrolysis of V^V^O_2_(acac)${}_{2}^{-}$. In the deconvoluted spectrum recorded in the system containing V^V^O_2_(acac)${}_{2}^{-}$ and lysozyme (Figure 7A), the signal corresponding to the mass of Lyz at 14304 Da and the series of signals of the adducts between the protein and one, two or three V^V^O${}_{2}^{+}$ ions are revealed. In all these adducts the acetylacetonato ligand is not coordinated to vanadium(V), confirming that the hydrolysis causes the removal of acac^−^ from the first coordination sphere of the metal; interestingly, the free V^V^O${}_{2}^{+}$ ion interacts with lysozyme which behaves as a polydentate ligand. Moreover, the adducts with one or two water ligands were revealed (peaks indicated by the asterisks in Figure 7).

The behavior of the system with ubiquitin is similar and the adducts *n*[V^V^O_2_]–Ub are detected with *n* = 1–3 (Figure 7B). This means that three sites of Ub are available for the metal binding.

**Figure 8:**
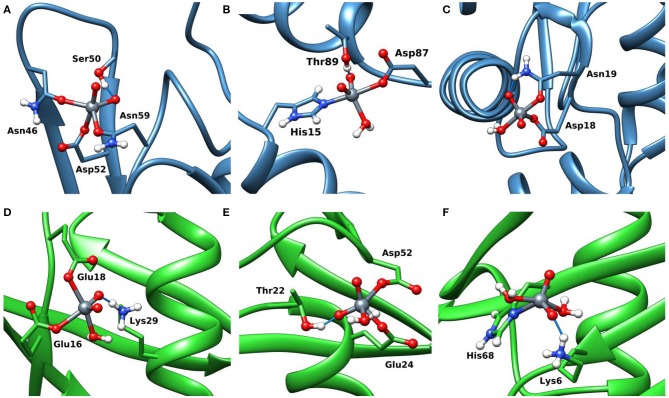
Most stable structures obtained by docking methods for the *active* binding of V^V^O${}_{2}^{+}$ to Lyz **(A–C)** and Ub **(D–F)**. The hydrogen bonds are highlighted with the full blue lines.

The docking results confirmed the suggestions of ESI-MS data and indicate that three sites are available both for Lyz and Ub. One, two or three donors are coordinated to V, which shows a slightly distorted trigonal bipyramidal environment (Figure 8). With lysozyme the binding occurs at site **A** (Asn46, Asp52, Asn59), **B** (His15, Asp87), **C** (Asp18, Asn 19) with different affinity and *F*_max_ of 48.5, 41.0, and 22.4 (Table 2). With ubiquitin the coordination to sites **1** (Glu16, Glu18) and **2′** (Glu24, Asp52) plus to His68 with *F*_max_ in the range 22.3–45.2 is predicted (Table 2). The monodentate coordination of Ub at His68 is stabilized by a V=O···H_3_N–Lys6 hydrogen bond and reminds that of His12 in acid phosphatase (Lindqvist et al., 1994).

**Table 2:** Docking solutions for the *active* binding of the V^V^O${}_{2}^{+}$ ion with Lyz and Ub.

**Protein**	**Site**	**Residues**	**V–donor^a^**	**2nd interaction**	**${\text{F}}_{\text{max}}^{\text{b}}$**	**${\text{F}}_{\text{mean}}^{\text{c}}$**	**Pop.^d^**	**Rank.^e^**
Lyz	A	Asn46, Asp52, Asn59	2.161, 2.157, 2.073	VO···Ser50	48.5	47.3	50/50	I
	A	Asn44, Asp52, Gln57	2.271, 2.415, 2.304	VO···Gln57	31.5	30.6	48/50	I
	B	His15, Asp87	2.095, 2.257	VO···Thr89	41.0	40.2	31/50	I
	C	Asp18, Asn19	2.196, 1.904	VO···Asn19	22.4	19.6	17/50	I
Ub	1	Glu16, Glu18	2.303, 2.309	VO···Lys29	45.2	44.5	49/50	I
	1'	Glu18, Asp21	1.958, 2.448	VO···Lys29	43.3	43.3	1/50	II
	2	Glu24, Asp52	2.915, 1.810	VO···Thr22	25.6	25.6	1/50	I
	2'	Glu51, Asp52	2.528, 2.213	VO···Lys27	25.2	24.9	19/50	II
	2'	Glu51, Asp52	2.368, 2.481	VO···Arg72	24.2	24.1	25/50	III
		His68	2.977	VO···Lys6	22.3	22.1	50/50	I

a *Distance between V and protein donors in Å*.

b *GoldScore Fitness value obtained for the more stable pose of each cluster (F_max_)*.

c *Average value of GoldScore Fitness for each cluster (F_mean_)*.

d *Population of the cluster*.

e *Ranking of the identified cluster*.

## Conclusions

Even though over the recent years many studies in bioinorganic and medicinal inorganic chemistry were dedicated to the development of vanadium-based potential drugs, the active oxidation state in the organism and mechanism of action are still elusive and their biospeciation has not completely explained. This lack of knowledge is one of the reasons why the number of V complexes under clinical tests is significantly lower than those of other metal ions. For example, while the oxidation state of Pt remains stable under physiological conditions (+II), for V three oxidation states are possible (+III, +IV, and +V) with an interconversion among them in the body. Therefore, in the design of vanadium drugs, such biotransformation and active state in the organism must be taken into account.

In this study, the V complexes with acetylacetonate, which displayed very promising antidiabetic and anticancer activity, were examined. It may be considered just as an example for the behavior of other V compounds. It was shown that, in the metal concentration range close to that found in the organism (15–250 μM), the species with +III state, V^III^(acac)_3_, is completely oxidized to V^IV^O(acac)^+^ and V^IV^O(acac)_2_ and this process is favored—with respect to the binary system without protein—by the binding of one or more amino acid side-chains. V^IV^O(acac)^+^ and V^IV^O(acac)_2_, in their turn, interact with proteins: up to four V^IV^O(acac)^+^ can bind to proteins to yield *n*[V^IV^O(acac)]–Ub/Lyz adducts, the binding of V^IV^O(acac)^+^ being favored with decreasing the vanadium concentration. At high concentration, also *n*[V^IV^O(acac)_2_]–Protein adduct may be revealed with an axial *active* or a surface *inert* interaction with the protein. The results with the complex in the +IV oxidation state are the same, with V^IV^O(acac)_2_ undergoing dissociation to the mono-chelated species V^IV^O(acac)^+^ moiety which binds to the proteins. Finally, V^V^O_2_(acac)${}_{2}^{-}$ undergoes complete dissociation to give the “bare” V^V^O${}_{2}^{+}$ ion. Docking calculations allowed us to predict the residues involved in the metal binding and three-dimensional structure of the formed adducts. Three important sites were predicted both for lysozyme and ubiquitin, **A** (with Asn44, Asn46, Asp48, Asp52, and Asn59), **B** (His15), and **C** (Asp18, Asn 19) for Lyz, and **1/1'** (Glu16, Glu18, Asp21), **2/2'** (Glu24, Glu51, Asp52), and His68 for Ub.

The results suggest that, at the studied conditions, only V^IV^O species of acetylacetonate survive in the presence of proteins and these could be the species responsible of the observed pharmacological activity. Starting from V^III^ or V^V^ may be useless, since V^III^ gives quantitatively V^IV^O, while V^V^ undergoes hydrolysis to the inorganic ions and the active species should be the same as when inorganic vanadium(V) was administered. This finding allow us to suggest that in this system and in those containing a bidentate organic ligand, V^IV^O ion should be used in the design of potential vanadium drugs, and that, if V^III^ or V^V^O_2_ potential active complexes had to be designed, species with high redox and thermodynamic stability should be synthesized to prevent oxidation and dissociation processes. To reach this aim, it is necessary to modulate adequately the features of the organic ligand and ligands with weak or intermediate strength such as acetylacetonate or maltolate should not be associated to V^III^ and V^V^ but only with V^IV^.

## Data Availability Statement

All relevant data is contained within the article. All datasets analyzed in this study are included in the article and the Supplementary Material.

## Author Contributions

EG conceived this study and designed the experiments and calculations. GS, SR, and J-DM performed the computational calculations. DS and GL performed the EPR measurements and VU the ESI-MS experiments. All authors contributed to the manuscript revision, read, and approved the submitted version.

## Conflict of Interest

The authors declare that the research was conducted in the absence of any commercial or financial relationships that could be construed as a potential conflict of interest.
