# Supplementary material for: Biospeciation of Potential Vanadium Drugs of Acetylacetonate in the Presence of Proteins
Source: Front Chem. 2020 May 7;8:345. doi: 10.3389/fchem.2020.00345 (PMC7221193; doi:10.3389/fchem.2020.00345)
Supplement: Supplementary file 1 [file Table_1.docx]

Supplementary Material


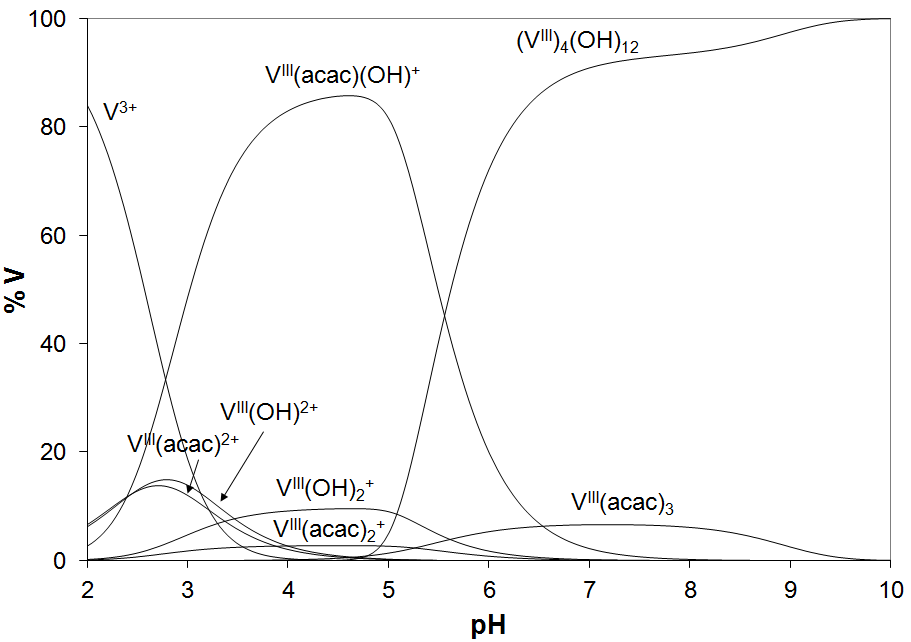


**Fig. S1**. Distribution diagram of the species as a function of pH in a solution containing V^3+^ ion and acac (V^III^/acac = 1/3, V^III^ = 1.50 × 10^–4^ M). The stability constants of the V^III^–acac complexes were taken from ([Brito et al., 2009](#_ENREF_1)).


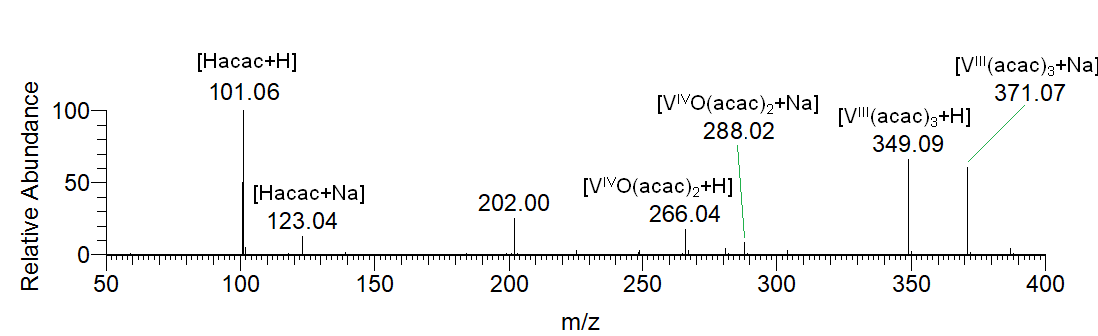


**Fig. S2**. ESI-MS spectrum recorded in ultrapure water on the systems containing V^III^(acac)_3_ with V concentration of 150 μM, pH 6.3. At these experimental conditions, V^III^ species partly oxidizes to V^IV^O.


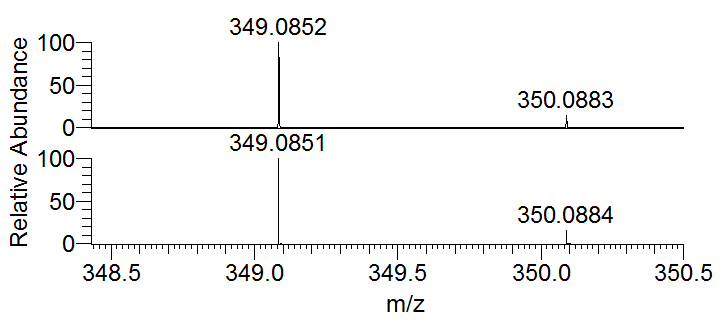


**Fig. S3**_._ Experimental (above) and calculated (below) isotopic pattern of the species [V^III^(acac)_3_+H^+^].


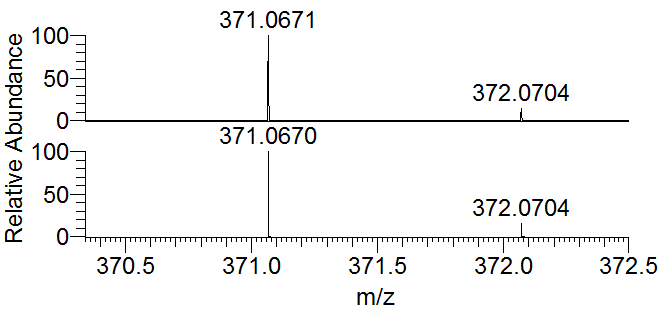


**Fig. S4**_._ Experimental (above) and calculated (below) isotopic pattern of the species [V^III^(acac)_3_+Na^+^].


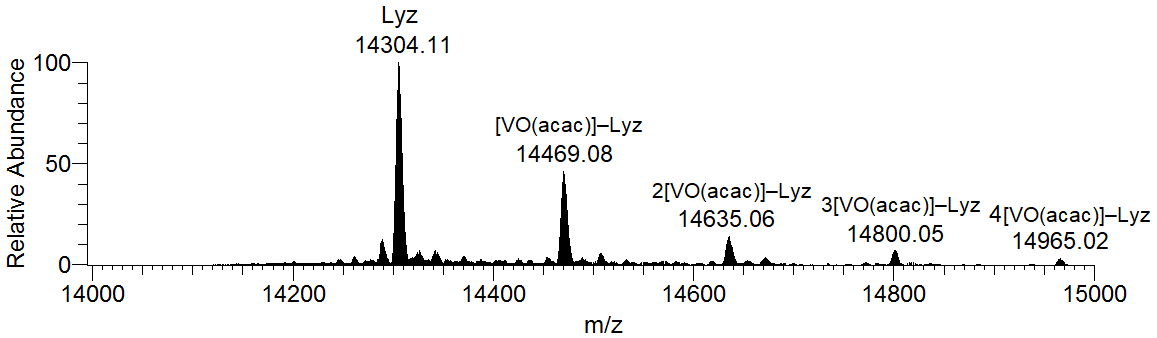


**Fig. S5**. Deconvoluted ESI-MS spectrum recorded on the systems containing V^III^(acac)_3_ and lysozyme with the molar ratio V^III^/Protein 3/1 and Protein concentration 5 μM, pH 6.5. Under these experimental conditions, V^III^ species fully oxidizes to V^IV^O


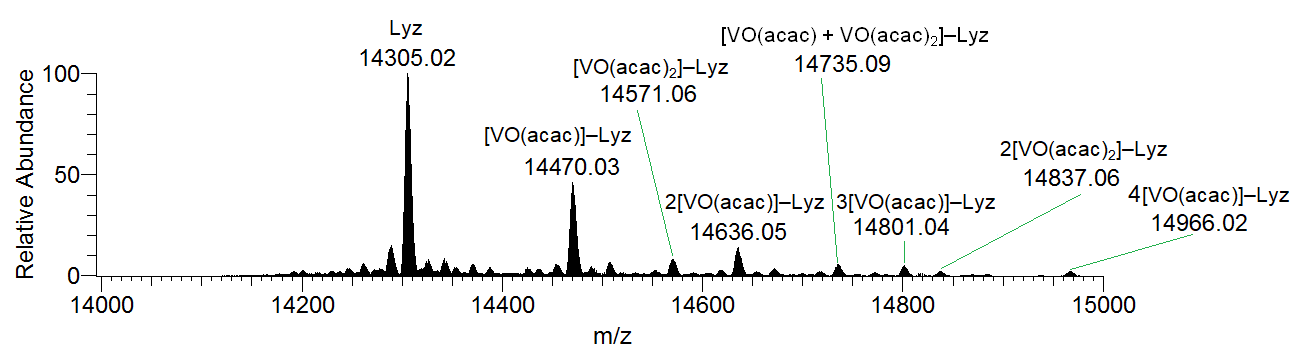


**Fig. S6**. Deconvoluted ESI-MS spectrum recorded on the systems containing V^IV^O(acac)_2_ and lysozyme with the molar ratio V^IV^/Protein 3/1 and Protein concentration 50 μM, pH 6.8.


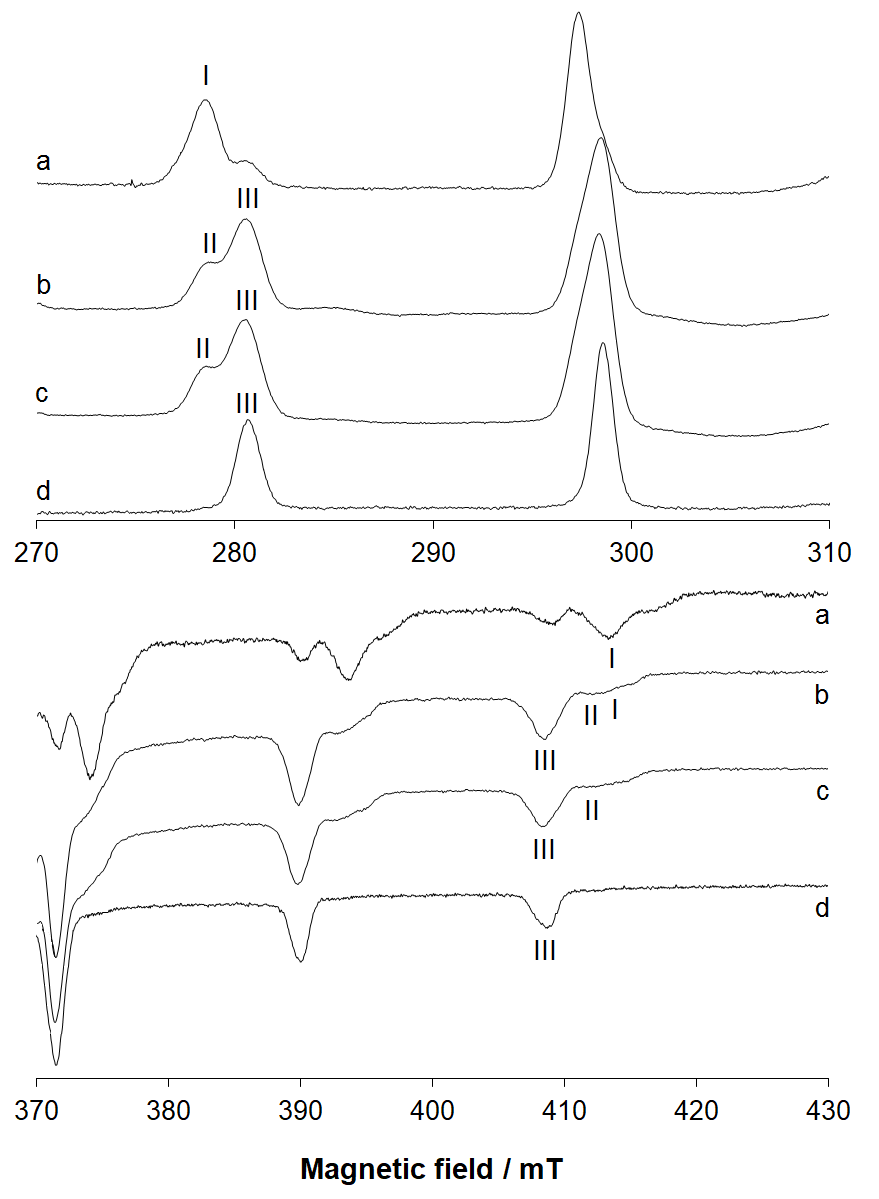


**Fig. S7**. Low and high-field region of the EPR spectra recorded on frozen solutions (120 K) of the systems: a) V^IV^O/acac 1/2, pH 4.40; b) V^III^(acac)_3_/Lyz 3/1, pH 4.95; c) V^III^(acac)_3_/Lyz 2/1, pH 5.05 and d) V^IV^O/acac 1/2, pH 5.75. V concentration was 1 mM. With **I**, **II** and **III** the *M*_I_ = -7/2, +7/2 resonances of the species V^IV^O(acac)^+^, [V^IV^O(acac)]–Lyz and [V^IV^O(acac)_2_]–Lyz, are indicated, respectively.


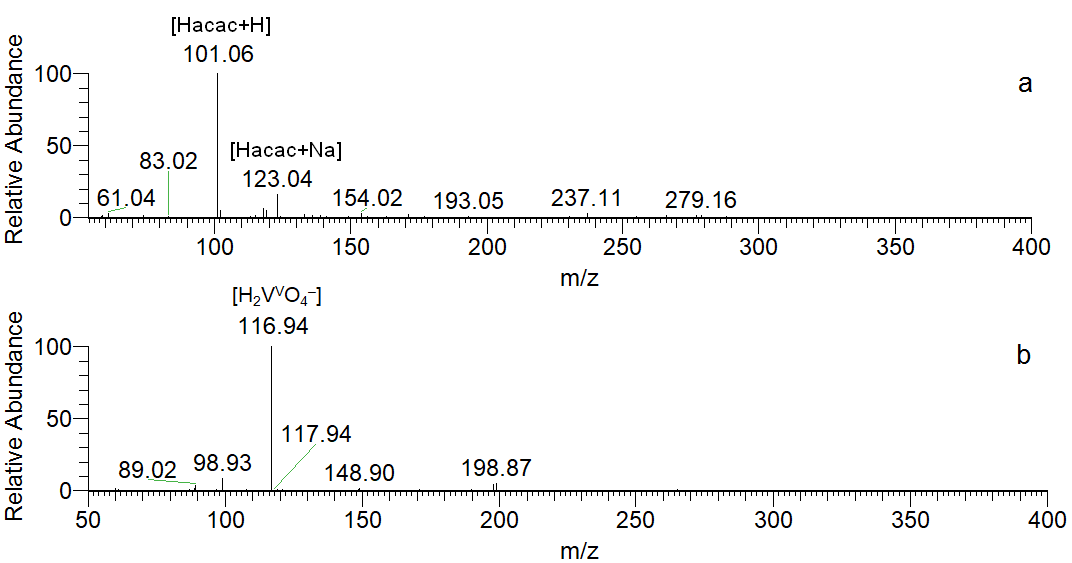


**Fig. S8**. ESI-MS spectra recorded in ultrapure water on the systems containing V^V^/acac with molar rario 1/2 and with V concentration of 150 μM, pH 7.0: a) positive-ion mode and b) negative-ion mode.

**References**

Brito, F., Araujo, M. L., Martínez, J. D., Hernández, Y., Moh, A., and Lubes, V. (2009). Speciation of the vanadium(III)–acetylacetone system in 3.0 M KCl ionic medium at 25°C. *J. Coord. Chem.* 62, 52-62. doi: 10.1080/00958970802474763.
